# Editorial: Emerging 3D and Animal Models in Diseases and Therapeutics

**DOI:** 10.3389/fmolb.2021.831833

**Published:** 2022-01-06

**Authors:** Yong Teng, Xiuping Yu, William C. Cho

**Affiliations:** ^1^ Department of Hematology and Medical Oncology, School of Medicine, Winship Cancer Institute, Emory University, Atlanta, GA, United States; ^2^ Department of Biochemistry and Molecular Biology, LSU Health-Shreveport, Shreveport, LA, United States; ^3^ Department of Clinical Oncology, Queen Elizabeth Hospital, Hong Kong SAR, China

**Keywords:** biomedical research, human diseases, translational sciences, 3D cell culture systems, animal models

Disease researchers have well studied the molecular and cellular properties of cell models propagated in two-dimensional (2D) culture. However, a lack of advanced *in vitro* and *in vivo* models has significantly impeded breakthroughs in understanding the fundamental mechanisms involved in diseases and thus, the development of new therapeutic strategies for patients. To change this situation, it has been deemed worthwhile to develop more physiologically relevant research models for mechanism-based target identification and drug discovery.

Recently, new technologies and the refinement of existing technologies in preclinical models have emerged. In particular, three-dimensional (3D) cell culture systems, which are used to model spheroids, organoids, and tissue heterogeneity, show promise, as they are more realistic and able to recapitulate the biological and physiological properties and functions of cultured cells and tissues. These new platforms, together with fit-for-purpose animal models, are key to success in bridging the gap between research and clinic.

Given this methodological advancement, we examine new opportunities for the development and application of medical research models in a range diseases and therapeutics and disseminate the latest findings in basic and translational research. In the present Research Topic, we have garnered several contributors to demystify novel 3D and animal models systems, explore the developments and challenges, and provide evidence-informed insights for facilitating enablers of knowledge translation.

Recently, the zebrafish (*Danio rerio*) have proven to be promising for the tumor microenvironment (TME) modeling. Teng’s team has performed pioneer studies using cost-effective zebrafish-cancer models (Teng et al., 2013; Shao et al., 2013; Xie et al., 2015; Xie et al., 2016; Shull et al., 2017). Combining different lines of discoveries in this cutting-edge field, this team systemically reviewed the recent ways zebrafish models have contributed to our understanding of cancer cell plasticity and tumor heterogeneity that modulated by TME (Loveless et al.). They also attempted to highlight how zebrafish models lend their utility to provide new insights into the various cellular and molecular mechanisms driving TME dynamics and tumor support. While zebrafish possess attractive advantages to assess tumor microenvironment interactions within the context of the immune system and later within the metastatic cascade, exploration into its capacity as tool to investigate TME interactions and drive patient therapies forward has only just begun.

There is no doubt that the mouse is the foremost mammalian model for studying human diseases and human health. In particular, genetically engineered mouse models have significantly contributed to our understanding of cancer biology and treatment response. It has also been well accepted that hormone-sensitive diseases are highly associated with age; thus, understanding how aging influences disease risk is quite important. To seek the answer, Liu and his group members established a novel virus-assisted spatially and temporally controlled Pten-null (referred to as *Pten* ^*adcre+*^) mouse model at different ages of adult mice (Liu et al.). This study provides a novel experimental model for developing age-related cancer, which enables us to further understand the effect of aging on carcinogenesis and identify pharmacological agents in the prevention and treatment of cancer in aged *vs.* non-aged mice.

Besides small animals, pigs represent a valuable animal for the development and validation of new medicines and procedures in human disease. The similarities of anatomy, physiology, metabolism, immunity, and genetics between pigs and humans make this model system informative for biomedical research, and the ability to experimentally induce tumors significantly broadens its application in cancer research. Of note, genetic alterations commonly found in human bladder cancer (BC), one of the costliest cancers to manage clinically, can be explored in genetically defined porcine models. In this sense, Segatto et al. outlined the currently available animal models of BC along with their limitations and proposed the swine as a fit-for-purpose animal to develop large humanized models of BC (Segatto et al.). Wu and his colleagues used immunosuppressed miniswine and successfully developed a suitable and paradigm-changing model to test the effects of various implant prototypes on reestablishing salivary functions (Wu et al.). These two large animal models are highly valuable to shape current demand and hold promise in the study of pathology and the screening of new therapeutic or diagnostic approaches for human diseases.

To meet an immense demand for highly efficient and easy methods for producing 3D spheroids, Ali et al. developed a straightforward procedure to form 3D spheroids from the newly isolated breast cancer KAIMRC1 cell line (Ali et al.). This procedure only requires growth media supplemented with 10% newborn calf serum and regular cell culture plates without the need for any specialized 3D cell culture system. de Poel et al. isolated crypts from rectal biopsies of subjects with cystic fibrosis (CF) and cultured them into intestinal organoids (de Poel et al.). As these organoids provide a relevant model in the context of preclinical drug discovery and lead selection, they used this model to evaluate the efficacy in the restoration of cystic fibrosis transmembrane conductance regulator (CFTR) among single, dual or triple combinations of ABBV/GLPG-2222, GLPG/ABBV-2737 and ABBV/GLPG-2451 to VX-809/VX-770. The resulting findings add value to this new model in defining personalized treatment regimes.

While it is tempting to envision new approaches for tissue modeling in normal and disease contexts given recent advances in gene editing and cellular reprogramming, we must recognize that no model is perfect. Different models serve for different purposes, though they may complement each other. With that said, biomedical research could easily go a step further by leveraging animal models when carefully selected, designed and utilized, which we believe is within reach. It is the right time to announce that a second edition of this topic is now live, which will continue to be an international platform for researchers to report and summarize the most recent developments and ideas with special emphasis on disease-related medical models.
